# Application of dGNSS in Alpine Ski Racing: Basis for Evaluating Physical Demands and Safety

**DOI:** 10.3389/fphys.2018.00145

**Published:** 2018-03-06

**Authors:** Matthias Gilgien, Josef Kröll, Jörg Spörri, Philip Crivelli, Erich Müller

**Affiliations:** ^1^Department of Physical Performance, Norwegian School of Sport Sciences, Oslo, Norway; ^2^St. Moritz Health and Innovation Foundation, Center of Alpine Sports Biomechanics, St. Moritz, Switzerland; ^3^Department of Sport Science and Kinesiology, University of Salzburg, Hallein, Austria; ^4^Department of Orthopedics, Balgrist University Hospital, University of Zurich, Zurich, Switzerland; ^5^Group for Snowsports, WSL - Institute for Snow and Avalanche Research SLF, Davos, Switzerland

**Keywords:** physical fitness, strength training, physical conditioning, external forces, air drag, ground reaction force, global navigation satellite systems, GPS

## Abstract

External forces, such as ground reaction force or air drag acting on athletes' bodies in sports, determine the sport-specific demands on athletes' physical fitness. In order to establish appropriate physical conditioning regimes, which adequately prepare athletes for the loads and physical demands occurring in their sports and help reduce the risk of injury, sport-and/or discipline-specific knowledge of the external forces is needed. However, due to methodological shortcomings in biomechanical research, data comprehensively describing the external forces that occur in alpine super-G (SG) and downhill (DH) are so far lacking. Therefore, this study applied new and accurate wearable sensor-based technology to determine the external forces acting on skiers during World Cup (WC) alpine skiing competitions in the disciplines of SG and DH and to compare these with those occurring in giant slalom (GS), for which previous research knowledge exists. External forces were determined using WC forerunners carrying a differential global navigation satellite system (dGNSS). Combining the dGNSS data with a digital terrain model of the snow surface and an air drag model, the magnitudes of ground reaction forces were computed. It was found that the applied methodology may not only be used to track physical demands and loads on athletes, but also to simultaneously investigate safety aspects, such as the effectiveness of speed control through increased air drag and ski–snow friction forces in the respective disciplines. Therefore, the component of the ground reaction force in the direction of travel (ski–snow friction) and air drag force were computed. This study showed that (1) the validity of high-end dGNSS systems allows meaningful investigations such as characterization of physical demands and effectiveness of safety measures in highly dynamic sports; (2) physical demands were substantially different between GS, SG, and DH; and (3) safety-related reduction of skiing speed might be most effectively achieved by increasing the ski–snow friction force in GS and SG. For DH an increase in the ski–snow friction force might be equally as effective as an increase in air drag force.

## Introduction

The physical demands on athletes in sport are primarily driven by the external forces acting in the interface between the athlete and the athlete's physical surroundings. In sport, the surroundings typically include the field of play where interaction forces occur between the athlete and the ground, sports apparatus (for example, high bars in gymnastics), sports gear (such as rackets in tennis) or fluids, such as air and water (water sports) (Knudson and White, 1989; Kolmogorov and Duplishcheva, 1992; Gastin et al., 2013). Hence, to quantify physical demands in sports, we first need to quantify the external forces acting on athletes. The validity of physical demand is therefore strongly related to the validity of the quantification of force. Validity of force measurement has two aspects; internal and external validity (Atkinson and Nevill, 2001). To maximize external validity the forces need to be captured in the natural sporting setting, preferably during competition, using measurement devices that provide minimal obstruction to athletes in the execution of their sport. Internal validity is achieved if precision and repeatability of force measurement is maximized. Alpine ski racing is an example of a sport that challenges both types of validity to a significant degree. The sport is executed in rough surroundings, athletes move at high speed over large distances (Kraemer et al., 2002; Kröll et al., 2016c), and safety and external validity aspects limit the force measurement equipment that can be mounted on athletes. Hence, the measurement of force is a difficult but important challenge in alpine skiing research and practice. Ground reaction forces are most commonly measured using pressure insoles or force plates (Mote, 1987; Lüthi et al., 2004; Federolf et al., 2008; Stricker et al., 2010; Nakazato et al., 2011; Kröll et al., 2016b; Falda-Buscaiot et al., 2017). Air drag force has been analyzed using wind tunnel testing (Luethi and Denoth, 1987; Savolainen, 1989; Thompson et al., 2001; Barelle et al., 2004; Meyer et al., 2011). However, to gain a holistic understanding of the external forces acting in skiing, these external forces need to be determined simultaneously and under field conditions. Since the measurement of ground reaction forces alone does not describe the entire physical demand, air drag force needs to be determined at the same time. Therefore, modeling has been applied to kinematic data to simultaneously derive air drag force and ground reaction forces in on-snow skiing for SL and GS (Brodie et al., 2008; Reid, 2010; Meyer et al., 2011; Supej et al., 2012; Gilgien et al., 2013). Such analysis has not so far been conducted for SG and DH, since methodologic limitations have not allowed for the measurement of skier kinematics over large capture volumes; hence, such knowledge is very limited in the speed disciplines (Gerritsen et al., 1996; Schiestl et al., 2006; Gilgien, 2014; Gilgien et al., 2014a, 2015a,b, 2016; Heinrich et al., 2014; Schindelwig et al., 2014; Yamazaki et al., 2015).

Recent advances in wearable measurement technology have allowed the reconstruction of skier kinematics across large capture volumes. These new methods combine differential global navigation satellite system technology (dGNSS) (Lachapelle et al., 2009; Andersson et al., 2010; Supej and Holmberg, 2011; Gilgien et al., 2014b) with digital terrain models (DTM) (Supej et al., 2012; Gilgien et al., 2013, 2015c; Nemec et al., 2014) or with inertial measurement technology (Brodie et al., 2008; Supej, 2010; Zorko et al., 2015; Fasel et al., 2016). Applying kinetic models to the captured kinematic data, both air drag force and ground reaction force and its components can be calculated simultaneously (Supej et al., 2012; Gilgien et al., 2013) without obstructing the athletes and thus ensuring high external validity (Atkinson and Nevill, 2001; Thomas et al., 2005), since skiers only wear a dGNSS unit on the body. This type of wearable technology allows the determination of skier kinematics and kinetics in skiing competitions across large capture volumes, such as entire SG and DH races, over several kilometers. The application of this new methodology is illustrated in computation of the physical demands with respect to adequate conditioning and an example taken from injury prevention for GS SG and DH.

### Physical demands and appropriate physical preparation

To prepare athletes for a certain sport the athlete's physical training needs to meet the coordinative affinity of the sport in competition (Muller et al., 2000). Specifically, the extent and magnitude athletes engage in static and dynamic muscular work and the nature of this muscular work need to correspond between training and competition. To ensure coordinative affinity between training and competition the prevalence, magnitude and the time–force pattern of the external forces need to be quantified and compared for the specific sport in training and competition. The physiological responses to alpine skiing in training and competition was assessed quite broad (Andersen and Montgomery, 1988; Neumayr et al., 2003; Turnbull et al., 2009; Ferguson, 2010). The scientific knowledge of the physical demands in alpine ski racing is limited to the technical disciplines slalom (SL) and giant slalom (GS) (Reid, 2010; Spörri et al., 2012b; Kröll et al., 2014, 2016c; Supej et al., 2014). Hence, to allow coaches and athletes to target their physical training specifically to the speed disciplines, the prevalence, magnitude and time–force patterns of the external forces need to be quantified for the speed disciplines SG and DH.

### External forces and injury prevention

The ability to withstand external forces in alpine ski racing is not only beneficial from a performance perspective (Raschner et al., 2012); if external forces exceed those an athlete's body can withstand, they lead to injuries. Therefore, the external forces acting in alpine skiing were not primarily examined with respect to physical demands on the athletes, but as a cause of injury (Mote, 1987; Bally et al., 1989; Quinn and Mote, 1992; Read and Herzog, 1992; Herzog and Read, 1993; Gerritsen et al., 1996; Yee and Mote, 1997; Hame et al., 2002; Raschner et al., 2012; Spörri et al., 2015). To prevent injuries, a good understanding is first needed of the contribution of external forces, and second of the consequences of changes in external factors, such as course setting and equipment, on external forces and injuries. Investigations were therefore conducted into how external forces are related to injury rates in the ski racing disciplines GS, super-G (SG) and downhill (DH) (Gilgien et al., 2014a), and how changes in ski geometry (Zorko et al., 2015; Gilgien et al., 2016; Kröll et al., 2016a,b; Spörri et al., 2016), course setting (Reid, 2010; Spörri et al., 2012c; Gilgien et al., 2014a, 2015a,b) and terrain (Supej et al., 2014; Gilgien et al., 2015a,b; Falda-Buscaiot et al., 2017) alter speed and external forces in these alpine skiing disciplines. However, one possibility for reducing speed and external forces, which was suggested by expert stakeholders in the ski racing community (Spörri et al., 2012a), was not investigated scientifically: increasing air drag by raising the air drag coefficient through changes in the materials used in athletes' clothing. An increase in air drag may increase the air drag force and the share of mechanical energy that is dissipated to the skier's surroundings, which in turn has the potential to lead to a reduction in skier speed (Bardal and Reid, 2014). Reduced skier speed might reduce the risk of injuries, especially in the case of high-impact accidents (Gilgien et al., 2014a, 2016). Therefore, we need to understand to what extent the braking forces in skiing, which are the air drag force and the ski–snow friction force, contribute to energy dissipation to the surroundings and subsequent speed reduction. Knowing the relative contributions of air drag and ski–snow friction forces to energy dissipation will allow us to understand whether an increase in air drag force or in ski–snow friction force is more effective in reducing speed and impact forces in accidents in each skiing discipline.

In the current study a new, validated and wearable dGNSS measurement-based method (Gilgien et al., 2013, 2015c) was applied to capture the external forces acting on forerunners skiing World Cup (WC) races in GS, SG and DH. The collected data were applied to illustrate the potential of such technology to enhance knowledge for scientists and practitioners on the physical demands of alpine skiing and injury prevention. For the first time, (i) the physical demands on the athletes in alpine skiing were assessed for GS, SG, and DH; and (ii) the effectiveness of energy dissipation and hence the ability to reduce skier speed was assessed for both air drag and ski–snow friction forces for GS, SG, and DH.

## Methods

### Measurement protocol

During the WC seasons 2010/11 and 2011/12, one male forerunner was equipped with a wearable dGNSS in various races. The forerunner was part of the official forerunner group and started directly prior to the respective WC races. Seven male WC giant slalom (GS) races—in total 14 runs—(Sölden (twice), Beaver Creek, Adelboden (twice), Hinterstoder, Crans Montana), 5 super-G (SG) races—in total 5 runs—[Kitzbühel, Åre, Hinterstoder, Crans Montana (twice)] and 5 downhill (DH) races—in total 16 runs including training runs—(Lake Louise, Beaver Creek, Wengen, Kitzbühel, Åre) were included in the analysis. In GS, each single competition run, and in DH, all training and competition runs were measured and analyzed. The forerunners were former male WC or current European Cup racers (age: 25.1 ± 3.6 years, mass: 86.1 ± 10.0 kg). This study was approved by the Ethics Committee of the Department of Sport Science and Kinesiology at the University of Salzburg and the athletes were informed of the investigation's purpose and procedures and signed written informed consent.

### Data collection methodology

**Figure 1:**
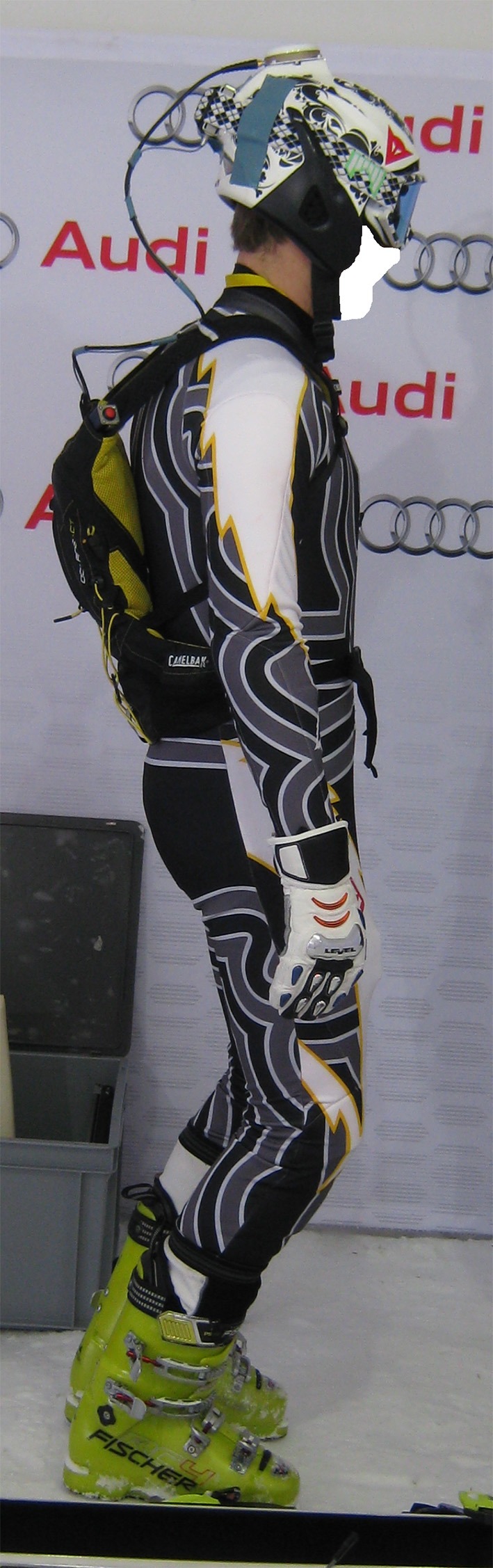
A forerunner equipped with a differential global navigation satellite system antenna on the helmet and a receiver in the cushioned backpack that was carried below a number bib during racing.

The forerunner's head trajectory was captured using kinematic dGNSS with the antenna (G5Ant-2AT1, Antcom, Canada) mounted on the helmet, and a GPS/GLONASS dual frequency (L1/L2) receiver (Alpha-G3T, Javad, USA) was carried in a small cushioned backpack (Figure 1). The total weight of the measurement equipment carried by the skier was 940 g (receiver 430 g, backpack 350 g, antenna 160 g). Differential kinematic carrier phase position solutions of the skier's trajectory were computed at 50 Hz using the data from two base stations consisting of antennas (GrAnt-G3T, Javad, USA) and receivers (Alpha-G3T, Javad, USA) mounted on tripods. The geodetic post-processing software GrafNav (NovAtel Inc., Canada) was used to compute differential kinematic carrier phase position solutions (Gilgien et al., 2014b).

The entire course width of the snow surface geomorphology was captured from start to finish using static dGNSS (Alpha-G3T receivers with GrAnt-G3T antenna, Javad, USA) and a Leica TPS 1230+ (Leica Geosystems AG, Switzerland). The number of points captured to describe the snow surface was dependent on the uniformity of the terrain. The less uniform the terrain, the more points were captured per area (in average on the entire course 0.3 points per m^2^). Based on the surveyed snow surface points a DTM was computed by applying Delaunay triangulation (de Berg et al., 2008) and smoothing using bi-cubic spline functions (Gilgien, 2014; Gilgien et al., 2015a,b).

### Parameter computation

#### Computation of the external forces

The antenna trajectory of the skier and the DTM were used as input parameters in a mechanical model (Gilgien et al., 2013) from which the ground reaction force (**F**_**SKI**_) and its component in the tangential direction to the skiers' trajectory (**F**_**SKI**_**-**_**FRICTION**_) were computed. The model also derived the air drag force (**F**_**AIR−DRAG**_). For a detailed description of the force computations (see Gilgien et al., 2013). **F**_**AIR−DRAG**_ was derived using body extension derived from the GNSS antenna position, a pendulum model attache to the antenna and the DTM, from skier speed which was derived from position data and a air drag cefficient model. The derivation of **F**_**SKI**_ and **F**_**SKI−FRICTION**_ was based on 1) the reconstruction of the center of mass position from the antenna position, the pendulum model attached to the antenna, and the DTM, 2) from the center of mass position the resultant force was calculated using time derivatives and mass of the athlete 3) **F**_**SKI**_ and **F**_**SKI−FRICTION**_ were calculated as the difference from the resultant force, **F**_**AIR−DRAG**_ and gravity.

### Characterization of the physical demands

For characterization of the physical demands, **F**_**SKI**_ was considered. The maximum **F**_**SKI**_ (**F**_**SKIMAX**_) was calculated for each turn as the average of the highest 10% of **F**_**SKI**_ for GS and SG, according to the method of Gilgien et al. (2014a). To approximate the fraction of time skiers were doing work in extended or crouched positions, the time in which skiers were skiing in a tucked position was approximated using the following criteria: (1) the CoM turn radius was larger than 125 m, and (2) the shortest distance from the GNSS antenna (which was mounted on the skier's helmet) position to the local terrain surface was less than the distance: 0.6 • body length + 6 cm. The time skiers were turning was defined as the periods when the CoM turn radius was smaller than 125 m. The time skiers were skiing straight but in an upright body posture (non-turning and non-tucked) was calculated as the difference between the sum of the time in tucked position and the time skiers were turning, as a percentage. CoM turn radius and distance to local DTM were computed according to the methods of Gilgien et al. (2015a,b,c).

To characterize the timing of **F**_**SKI**_ through a turn cycle in GS and SG for each turn and averaged across all turns, the time for the following sections (phases) were calculated: from turn transition at the beginning of the turn (switch1) to gate passage; from switch1 to the time of **F**_**SKIMAX**_; from gate passage to turn transition at the end of the turn (switch2); and the overall turn cycle time (from switch1 to switch2). Turn transition was calculated as the deflection point of the CoM trajectory between turns (Gilgien et al., 2015a,b). Run time is a rough estimation of total workload, while impulse (the integration of air drag and ground reaction force over the run time), is a measure of the total workload. Impulse and run time were calculated according to the methods of Gilgien et al. (2014a).

### Contribution of external forces to energy dissipation

The instantaneous energy dissipation due to ski–snow friction, EDISS_SKI_ and energy dissipation due to air drag, EDISS_AIR_ were computed according to Equations (1) and (2). The relative contributions of EDISS_SKI_ and EDISS_AIR_ to the total instantaneous energy dissipation (sum of EDISS_SKI_ and EDISS_AIR_) were expressed as percentages of total instantaneous energy dissipation.

(1) $$EDISS_{SKI}=\;\int F_{SKI-FRICTION}(t)v(t)dt$$

(2) $$EDISS_{AIR}=\;\int F_{AIR-DRAG}(t)v(t)dt$$

### Statistical analysis

Normality of instantaneous data from all races in each discipline was tested using a Lilliefors test (α = 0.05). No parameter was found to be normally distributed, so non-parametric statistics were applied to compare all parameters between disciplines. Median and inter-quartile range (IQR) were computed for all parameters and disciplines. The relative sizes of parameters for GS and SG compared to DH were computed from the medians of each discipline and were expressed as percentages of DH medians. In addition, mean and standard deviation were calculated for the time skiers were in tucked position, the time skiers were turning, the time spent skiing in non-turning and non-tucked position, impulse, and run time for all disciplines. The medians of the disciplines were tested using an ANOVA, Kruskal–Wallis test (*p* = 0.01), followed by a Friedman's test (*p* = 0.01) if significant differences were found in the ANOVA.

For GS and SG mean and standard deviations were also computed for turn cycle time characteristics, number of direction changes and **F**_**SKIMAX**_. For **F**_**SKI**_, turn cycle means were computed for each 10% increment of the time-normalized turn cycles for SG and GS.

## Results

### Overview of external forces

**Figure 2:**
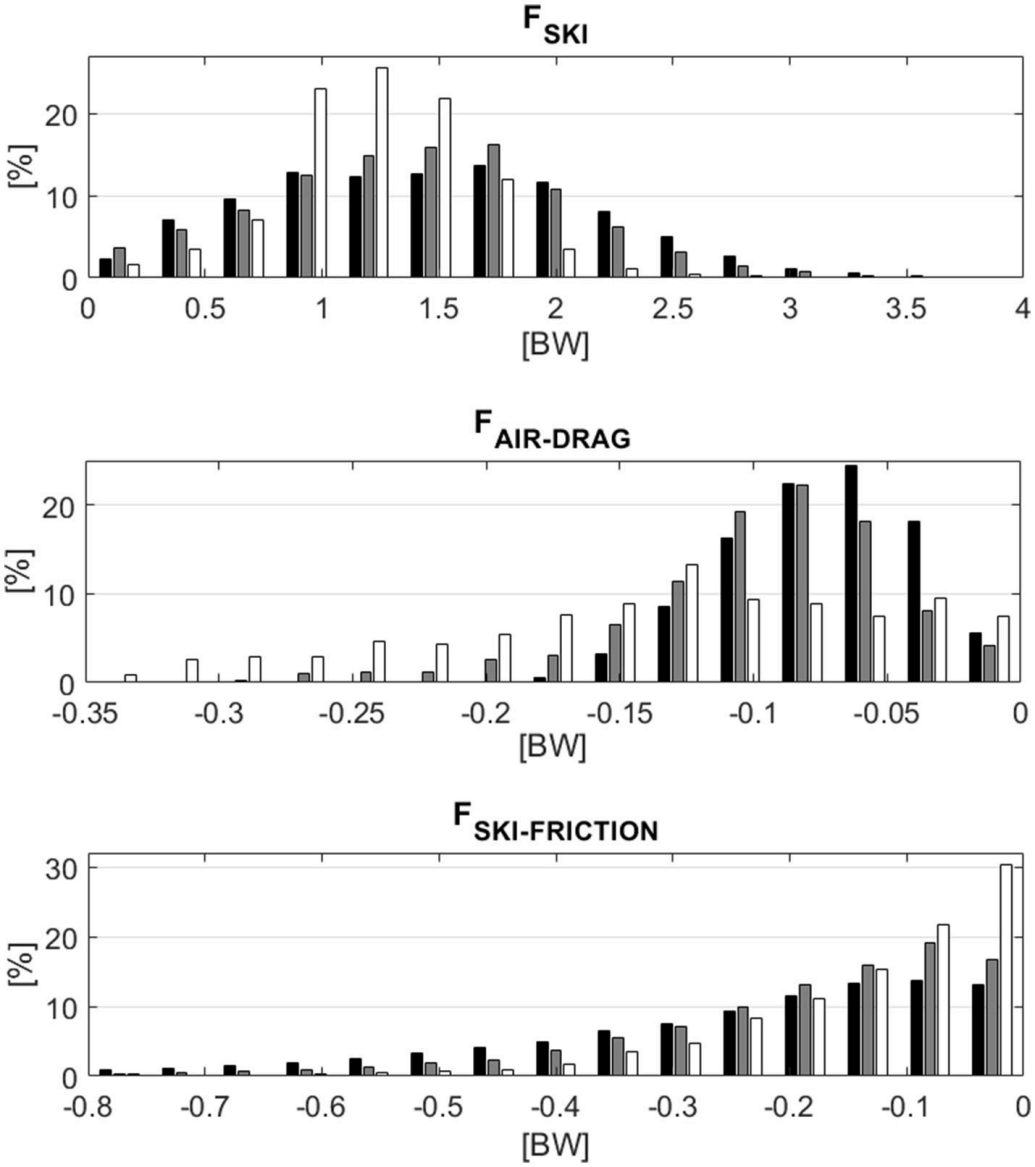
Histograms of the force distributions within and between disciplines for ground reaction force (**F**_**SKI**_), air drag force (**F**_**AIR−DRAG**_), and ski–snow friction force (**F**_**SKI−FRICTION**_). Giant slalom is plotted in black, Super-G in gray and Downhill in white.

The median, IQR and the percentage values for GS and SG in relation to DH are given in Table 1. The medians were significantly different (*p* = 0.01) between disciplines for all forces. Figure 2 illustrates the differences in forces [expressed in body weight ([BW)] between disciplines in histograms. The median **F**_**SKI**_ was 22% larger for GS and 15% larger for SG compared to DH. The IQRs were largest for GS, followed by SG and DH. In GS and SG skiers skied for about 40% of the time with **F**_**SKI**_ values larger than 1.5 BW, while in DH values above 1.5BW were achieved for less than 20% of the time. **F**_**SKI−FRICTION**_ median was doubled for GS compared to DH and 52% larger for SG compared to DH. The IQR was largest for GS, followed by SG and DH. The median **F**_**AIR−DRAG**_ was largest for DH, followed by SG and GS, and was approximately twice as large for DH as for GS. IQR was largest for DH, followed by SG and GS. In DH, **F**_**AIR−DRAG**_ was larger than 0.2 BW for ~25% of the time, while this magnitude occurred for less than 2% of the time in GS.

**Table 1:** Median and interquartile range (IQR) of the absolute values for all disciplines and the relative values for Giant slalom and Super-G compared to Downhill.

	**Absolute values median** ± **IQR**			% of DH^*^	
	**GS**	**SG**	**DH**	**GS**	**SG**
F_SKI_ [BW]	1.46 ± 1.04	1.42 ± 0.86	1.21 ± 0.53	122	115
F_SKI−FRICTION_ [BW]	0.20 ± 0.27	0.15 ± 0.19	0.10 ± 0.15	202	152
F_AIR−DRAG_ [BW]	0.07 ± 0.05	0.09 ± 0.06	0.13 ± 0.12	57	71

* *The value of DH is equal to 100%*.

*F_SKI_ (ground reaction force), F_AIR−DRAG_ (air drag force), F_SKI−FRICTION_ (ski – snow friction)*.

### Characterization of the physical demands

The measures for total load on athletes, run time and impulse had showed the highest values for all measures in DH, followed by SG and GS (Table 2). The percentage of total run time in which athletes were turning was longest in GS, followed by SG and DH. The total time athletes were in tucked position was longest in DH, followed by SG and GS. The time when skiers were not turning and were in an upright position did not differ between disciplines. For results see Table 2.

**Table 2:** Mean and standard deviation for run time, impulse per run; percentage of time skiers are turning per run; percentage of time skiers are not turning but are not in tucked position per run; percentage of time skiers are in tucked position per run for all disciplines.

		**Run time [s]**	**Impulse [kBWs]**	**Time turning [%]**	**Time non—turning and non—tucked [%]**	**Time in tucked position [%]**
Giant slalom	Mean	77.4	124.3	92.80	5.40	1.80
	SD	5.20	12.5	2.1	2.1	2.1
Super-G	Mean	92.90	153.0	79.37	4.43	16.20
	SD	9.70	13.3	6.5	6.5	6.5
Downhill	Mean	121.4	173.4	54.84	8.36	36.80
	SD	17.7	25.3	8.1	8.1	8.1

**Figure 3:**
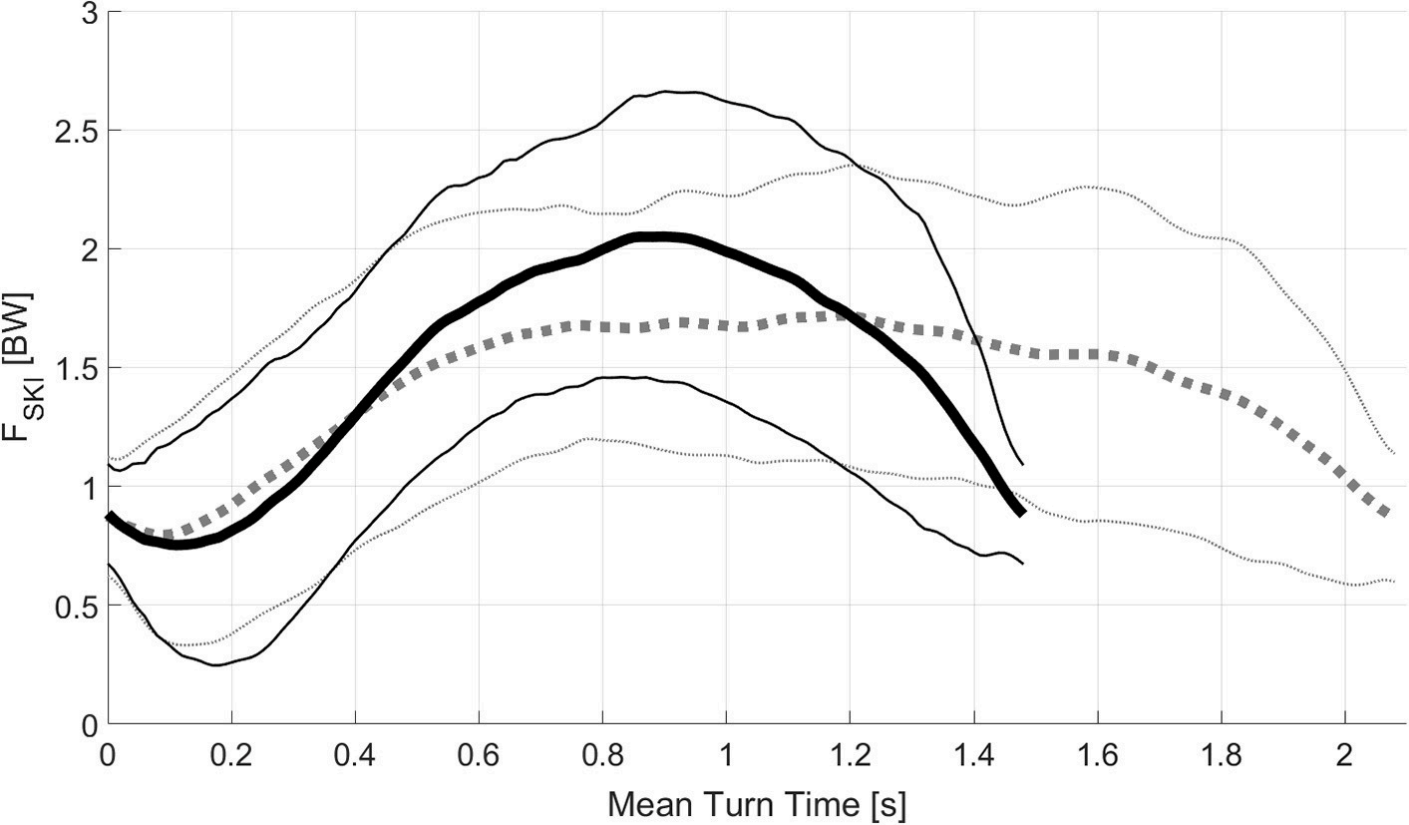
Turn cycle characteristics for ground reaction force (**F**_**SKI**_) for Giant slalom in black and Super-G in gray as a function of mean turn cycle time. Instantaneous mean in solid line, Standard deviations in thin line.

An SG run consisted of 41 turns, while GS consisted of 51 turns, which indicates that SG consists of a highly cyclic turn pattern where skiers turn for 79.4% of the run time while in GS they turn for 92.8% of the run time (Table 2). Force-time characteristics are illustrated in Figure 3 and Tables 3, 4. Figure 3 shows the **F**_**SKI**_ and COM turn radius as a function of mean turn time for GS and SG with the mean drawn in solid lines and standard deviations in dashed lines for **F**_**SKI**_. To allow quantitative reconstruction of the **F**_**SKI**_—turn cycle time relationships in GS and SG these are provided as 10% turn cycle time increments in Table 3. Turn timing characteristics, along with the number of direction changes and **F**_**SKIMAX**_ characteristics, are provided in Table 4.

**Table 3:** Mean and standard deviation for ground reaction force in BW for 10%-wise increments of the turn cycle for Giant slalom and Super-G.

**% of turn cycle**		**0–10**	**10–20**	**20–30**	**30–40**	**40–50**	**50–60**	**60–70**	**70–80**	**80–90**	**90–100**
GS	Mean	0.79	0.86	1.21	1.62	1.86	2.00	2.01	1.86	1.60	1.15
	SD	0.33	0.54	0.51	0.51	0.48	0.52	0.58	0.62	0.62	0.38
SG	Mean	0.85	1.13	1.49	1.65	1.68	1.70	1.64	1.55	1.42	1.09
	SD	0.39	0.51	0.53	0.46	0.49	0.56	0.56	0.64	0.60	0.42

**Table 4:** Ground reaction force and turn cycle characteristics for Giant slalom and Super-G.

		**Time switch1 to Gate [s]**	**Time gate to switch2 [s]**	**Turn cycle time [s]**	**Time point of F_SKIMAX_ [s]**	**F_SKIMAX_ [BW]**	**Number of direction changes**
GS	Mean	0.87	0.60	1.47	0.86	3.16	51.2
	SD	0.30	0.25	0.41	0.06	0.72	3.5
SG	Mean	1.20	1.07	2.28	1.23	2.79	40.8
	SD	0.44	0.51	0.73	0.15	0.57	4

### Contribution of external forces to energy dissipation

For the dissipative forces **F**_**AIR−DRAG**_ and **F**_**SKI−FRICTION**_, median energy dissipation to the surroundings was not significantly different between GS and SG for EDISS_SKI_. All other skiing discipline median values were significantly different between disciplines for both energy dissipation types (Table 5). The median EDISS_SKI_ was 41% (GS) and 42% (SG) larger than for DH. The median for EDISS_AIR_ was found to be 41% (GS) and 71% (SG) of the median for DH. DH had also the largest IQR. The relative contributions of energy dissipation (median) due to air drag and ski–snow friction were found to be 23% (EDISS_AIR_) and 77% (EDISS_SKI_) in GS, 35% (EDISS_AIR_) and 65% (EDISS_SKI_) in SG and 51% (EDISS_AIR_) and 49% (EDISS_SKI_) in DH.

**Table 5:** Median and interquartile range (IQR) of the absolute values for all disciplines and the relative values for Giant slalom and Super-G compared to Downhill.

	**Absolute values Median** ± **IQR**			% of DH^*^	
	**GS**	**SG**	**DH**	**GS**	**SG**
EDISS_SKI_ [BW·m]	−0.07 ± 0.09	−0.07 ± 0.09	−0.05 ± 0.08	141	142
EDISS_AIR_ [BW·m]	−0.02 ± 0.02	−0.04 ± 0.03	−0.06 ± 0.07	41	71

* *The value of DH is equal to 100%*.

*EDISS_SKI_ (energy dissipation due to ski–snow friction), EDISS_AIR_ (energy dissipation due to air drag force)*.

**Figure 4:**
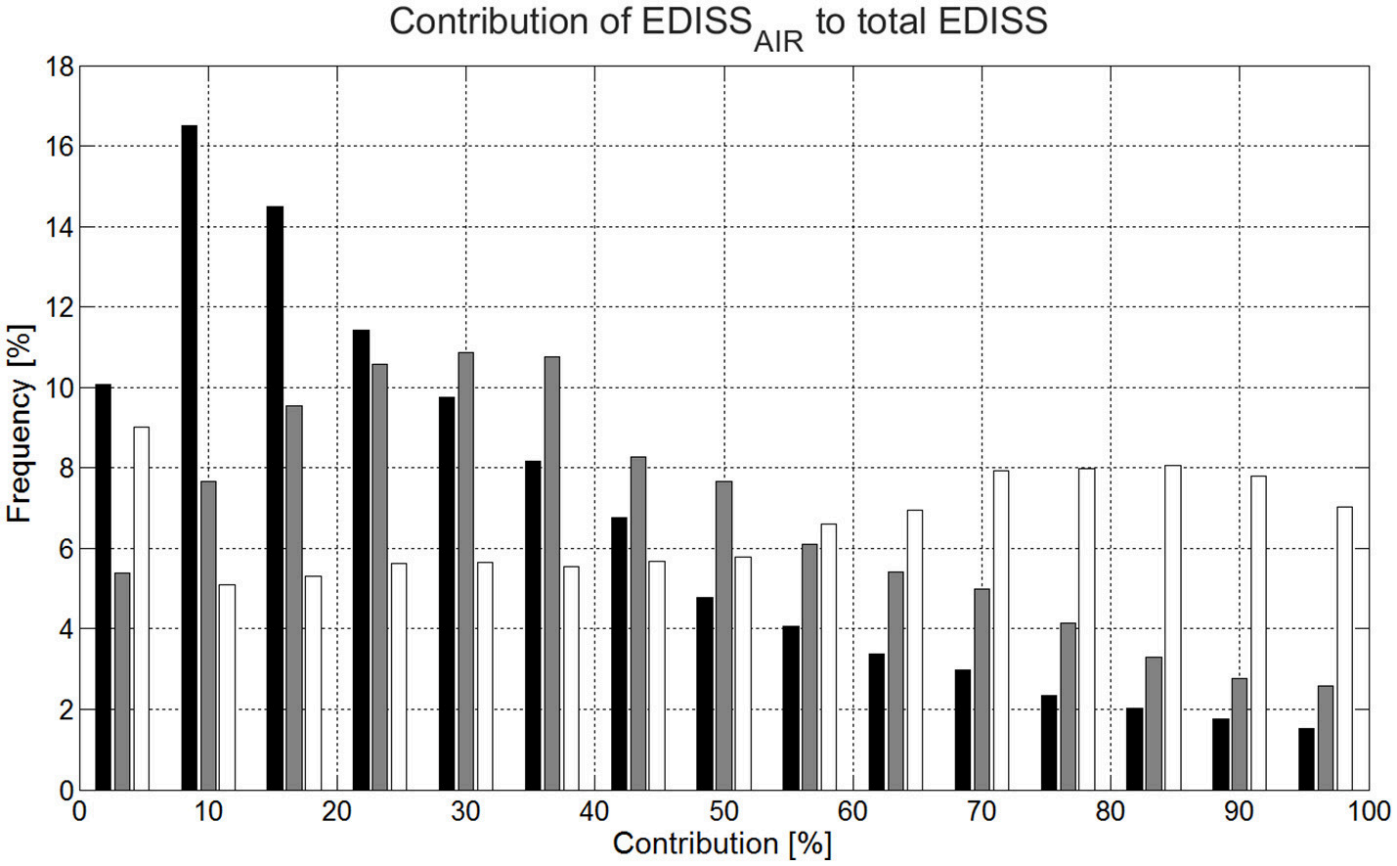
Histogram illustrating the percentage contribution of air drag to total energy dissipation for Giant slalom, Super-G and Downhill. Giant slalom is plotted in black, Super-G in gray and Downhill in white. The horizontal axis shows the contribution of energy dissipation due to air drag as a percentage of total energy dissipation, while the vertical axis shows how often these contributions were present (frequency).

Figure 4 illustrates the relative contribution of **F**_**AIR−DRAG**_ and **F**_**SKI−FRICTION**_ to the total energy dissipation (EDISS) as a percentage contribution of EDISS_AIR_ to total energy dissipation for GS, SG and DH. The horizontal axis shows the contribution of EDISS_AIR_ as a percentage of total energy dissipation, while the vertical axis shows the frequency of occurrence of these contribution patterns. The percentage contribution of EDISS_SKI_ to total energy dissipation was complementary to the percentage contribution of EDISS_AIR_ to total EDISS, since **F**_**SKI−FRICTION**_ and **F**_**AIR−DRAG**_ are the only sources for EDISS. For more than 80% of the time EDISS_SKI_ had a larger contribution to total EDISS than EDISS_AIR_ in GS, while in DH the contribution of EDISS_SKI_ was larger than the contribution of EDISS_AIR_ to total EDISS for less than 40% of the run time.

## Discussion

The study revealed that: (1) the method was effectively applied to capture external force data from WC races; (2) the physical demands in alpine ski racing were mainly characterized by fluctuations in the ground reaction force, which followed a cyclic pattern and was most pronounced for GS, followed by SG and DH; and (3) injury prevention measures using an increase in air drag would be about equally effective as measures that cause an increase in ski–snow friction for DH, while for GS and SG measures that cause an increase in ski–snow friction would be most effective.

### The application of dGNSS technology to capture external force data from WC races in alpine skiing

It has been shown that if high-end dGNSS devices are carefully applied, antenna position accuracy to less than 5 cm can be reached even in highly dynamic sports such as alpine skiing (Gilgien et al., 2014b, 2015c). It has also been shown that the position accuracy of a dGNSS allows valid derivation of velocity and of the external forces acting on skiers simultaneously (Gilgien et al., 2013, 2015c). The present study showed that the high validity of the wearable technology allowed detailed investigation of aspects of physical fitness and injury prevention that are relevant for practitioners of a sport where athletes move at high speed through rough surroundings and over large distances. Also, the method proved to be valid and practicable to be applied in a large number of WC races.

### Characterization of the physical demands

To get a rough idea of the physical demands of a sport or a discipline the total physical load may serve as a good indication. Run time provides limited information, since the intensity of the work done is not measured. Measuring impulse—the integration of the external forces **F**_**AIR−DRAG**_ and **F**_**SKI**_ over the run time—might describe the total load better. Comparing the three disciplines, impulse was highest in DH, followed by SG and GS if only one run was considered in GS (Gilgien et al., 2014a). In GS, athletes actually ski two runs, if they qualify for the second run. Hence, the impulse for the first run, the 3 h break between the two runs and the warm-up to the second run define the demands for physical recovery between runs for that discipline.

To understand the total physical load on athletes in more detail, we need to compare the factors contributing to the impulse. These are run time, **F**_**AIR−DRAG**_ and **F**_**SKI**_. Run time was longest in DH followed by SG and GS, while the sum of median **F**_**AIR−DRAG**_ and **F**_**SKI**_ was highest for GS (1.53 BW), followed by SG (1.51 BW) and DH (1.34 BW). Hence, despite the higher external forces in GS and SG compared to DH, run time seems to have a major impact and lead to higher impulses and total physical loads per run for the speed disciplines compared to GS.

Comparing the type of work between the disciplines there is an obvious difference between GS and SG compared to DH. Inspecting the histogram for **F**_**SKI**_ in Figure 2, **F**_**SKI**_ is overrepresented in the small and high force ranges for GS and SG compared to DH. This might be a consequence of more pronounced repeated loading-unloading patterns and higher peak forces in GS and SG compared to DH. DH consists of longer sections of straight skiing, while SG and GS consist of more or less continuous turning. In GS, skiers turned for 92.8% of the run time, in SG for 79.4% of the run time, and in DH skiers turned for only 54.8% of the run time (Table 2). These differences in the amount of direction alteration in skier trajectory are reflected in the higher median **F**_**SKI**_ for GS and SG compared to DH, and also indicate substantial differences in the type of physical work athletes conduct in the different disciplines. GS consists of 51 direction changes (Table 4), meaning that GS involves 51 body extension-contraction cycles, while SG consists of 41 direction changes and extension-contraction cycles. Therefore, GS and SG consist of more or less continuous turning and dynamic muscular work, while in DH skiers ski straight for about 45% of the run time and spend 36.8% of the run time in a tucked position (Table 2). The amount of skiing in the tucked position might be a consequence of both the extent of sections in which skiers can ski straight, and also the higher speed compared to the other disciplines, which increases the significance of air drag force as a dissipative force (Table 5 and Figure 4). Therefore, skiers try to reduce the time skiing in upright body posture, since this is likely to increase the drag area exposed to wind and increase air drag (Barelle et al., 2004; Supej et al., 2012). Hence, in DH skiers try to reduce speed loss through energy dissipation by air drag force. Because of the lower number of direction changes, skiers spend more of the total run time in the tucked position undertaking work of a more static nature with less pronounced and less frequent unloading phases, over a longer period compared to GS for instance. An earlier comparative study on SG, GS and SL revealed that a more static nature of movement in SG results in deeper knee angle and is accompanied with significantly higher EMG activity (Berg and Eiken, 1999) compared to GS and SL. While the EMG activity during SG depicted for the quadriceps muscle values of 120% muscular voluntary contraction in GS and SL only values in the order of 70% MVC were observed. Hence, tucked body position is associated with more static muscular work and increased muscular activity.

Comparing GS and SG, which consist of more or less consecutive turning (Gilgien et al., 2014a, 2015a,b), with 51 turns, or 51 loading-unloading cycles in GS compared to 41 in SG, the duration of an average turn cycle in SG (2.28 s) is about 55% longer than in GS (1.47 s). However, mean **F**_**SKI**_ and **F**_**SKIMAX**_ are lower in SG compared to GS. Therefore, in SG athletes need to withstand a lower **F**_**SKI**_ but over a longer period of time. Figure 3 shows that the mean **F**_**SKI**_ is larger than 1.5 BW for 1.18 s (from 0.50 to 1. 68 s after switch1) in SG, while in GS mean **F**_**SKI**_ is larger than 1.5 BW for 0.81s (from 0.47 to 1. 31 s after switch1). In short, in SG athletes need to withstand a force larger than 1.5 BW for 0.37 s longer than in GS. In both disciplines, **F**_**SKIMAX**_ occurs at gate passage and the time from turn initiation (switch1) to gate and time to the occurrence of **F**_**SKIMAX**_ is longer than from gate to turn completion (switch2). This means that building up the maximal force occurs over a longer period of time than turn completion for both disciplines. The time to build up **F**_**SKIMAX**_ is substantially shorter in GS compared to SG. Therefore, athletes face a substantially more pronounced loading–unloading pattern than in SG, with a higher **F**_**SKI**_ but a shorter time to the next unloading phase. The loading–unloading pattern is even more pronounced in slalom, where the loading–unloading time is shortest and highest **F**_**SKI**_ compared to the other disciplines (Reid, 2010; Kröll et al., 2016a). These substantial differences in **F**_**SKI**_ characteristics between probably need different physical preparation to maximize performance and minimize injury risk. The **F**_**SKI**_ and turn cycle timing information might be useful for coaches and athletes in adapting dryland training to the discipline-specific **F**_**SKI**_–time pattern, since dryland training that simulates the physical demands of competitive skiing might lead to an adequate physiological adaptation (Kraemer et al., 2002; Kröll et al., 2016c). In order to imitate the physical demands of alpine skiing in dryland training, skiing simulators (Nourrit et al., 2003; Deschamps et al., 2004; Hong and Newell, 2006; Teulier et al., 2006; Panizzolo et al., 2013; Moon et al., 2015; Lee et al., 2016) and skiing carpets (Fasel et al., 2017) are used to a certain extent. The data provided in this study might help to adapt these devices to the physical demands of competitive on-snow skiing with respect to the discipline-specific force–time pattern.

### Contribution of external forces to energy dissipation

The analysis of the dissipative forces contributing to total EDISS (Figure 4) confirmed the finding from another study (Supej et al., 2012) that EDISS in GS is mainly determined by **F**_**SKI−FRICTION**_. In SG, **F**_**SKI−FRICTION**_ was still clearly the major contributor to EDISS, while the contributions of **F**_**SKI−FRICTION**_ and **F**_**AIR−DRAG**_ were approximately balanced in DH. Hence, for slalom (Reid, 2010), GS and SG, a certain percentage increase of **F**_**SKI−FRICTION**_ would have a larger effect on performance than a corresponding increase of **F**_**AIR−DRAG**_, while in DH the effect of an increase in the dissipative forces, **F**_**SKI−FRICTION**_ and **F**_**AIR−DRAG**_ by an increase in air drag coefficient through clothing would be about equal. Comparing DH with speed skiing, the contribution of **F**_**AIR−DRAG**_ to total EDISS seems clearly smaller in DH than in the discipline speed skiing, where skiers do not turn, but ski straight along the fall line to reach maximal speed, **F**_**AIR−DRAG**_ contributes up to 80% of total EDISS when maximal speed is reached (Thompson et al., 2001). Hence, for the alpine ski racing disciplines, an increase in **F**_**AIR−DRAG**_ might only be an option for DH.

## Limitations

One potential drawback of the applied method is that ground reaction forces cannot be determined for single legs, but only for the sum of both legs. In addition, high frequency force components cannot be determined with the method used in this study. However, the method was chosen since it allows the measurement of all external forces and their components at the same time, allowing unique insight in their relationship as shown in this study. The applied method does not measure, but rather models the external forces based on kinematic data and was validated against the gold standard for GS (Gilgien et al., 2013, 2015c). Therefore, comparison of the findings from this study with previous findings reported in the literature, where forces were obtained with other methods, are of interest with respect to validity. An experimental GS study using a video-based photogrammetric method to compute skier kinematics, from which forces were derived in steep terrain (26°), found mean turn **F**_**SKI**_ s values of between 1.52 and 1.56 BW (Spörri et al., 2016). The maximal **F**_**SKI**_ values found in that study ranged from 2.01 to 2.11 BW (Spörri et al., 2016), while a comparable study in 23° inclined terrain found a range of 2.32–2.44 BW for the maximal **F**_**SKI**_ using pressure insoles to measure **F**_**SKI**_ (Kröll et al., 2016a). Comparing these **F**_**SKI**_ values with the **F**_**SKI**_ values obtained in the current study for GS, we conclude that the **F**_**SKI**_ values are comparable to those found for competitive skiing in previous studies and obtained with different methods. This finding increases confidence in the kinetic method applied in this study for SG and DH, where no **F**_**SKI**_ data are available in the literature with which to compare our results for SG and DH. The applied method does not allow to analyze the distribution of **F**_**SKI**_ between legs. This might be interesting for the speed disciplines, since previous studies found that the distribution changes from SL to GS (Kröll et al., 2016c).

## Conclusion

This study (1) illustrated that the validity of high-end dGNSS systems allows meaningful investigations such as characterization of physical demands and safety measures in highly dynamic sports; and (2) showed that the physical demands were substantially different between GS, SG and DH (specifically, the ground reaction force fluctuations followed a cyclic pattern, which was most pronounced for GS, followed by SG and DH, while median and peak ground reaction forces were highest for GS, followed by SG and DH); and (3) revealed that safety-related reduction of skiing speed might be most effectively achieved by increasing the ski–snow friction force in GS and SG. For DH an increase in the ski–snow friction force might be equally as effective as an increase in air drag force.

## Author contributions

MG, JK, JS, and EM designed the study. MG, JK, JS collected the data. MG and PC analyzed the data. All authors contributed to the writing.

### Conflict of interest statement

The authors declare that the research was conducted in the absence of any commercial or financial relationships that could be construed as a potential conflict of interest.
